# VEGF-R2/Caveolin-1 Pathway of Undifferentiated ARPE-19 Retina Cells: A Potential Target as Anti-VEGF-A Therapy in Wet AMD by Resvega, an Omega-3/Polyphenol Combination

**DOI:** 10.3390/ijms22126590

**Published:** 2021-06-19

**Authors:** Flavie Courtaut, Alessandra Scagliarini, Virginie Aires, Clarisse Cornebise, Jean-Paul Pais de Barros, Céline Olmiere, Dominique Delmas

**Affiliations:** 1 Université de Bourgogne Franche-Comté, 21000 Dijon, France; flavie.courtaut@gmail.com (F.C.); alescaglia@gmail.com (A.S.); virginie.aires02@u-bourgogne.fr (V.A.); clarisse.cornebise@gmail.com (C.C.); jppais@u-bourgogne.fr (J.-P.P.d.B.); 2 INSERM Research Center U1231—Cancer and Adaptive Immune Response Team, Bioactive Molecules and Health Research Group, 21000 Dijon, France; 3 Lipidomic Analytical Platform, 21000 Dijon, France; 4 Laboratoires Thea, 12 Rue Louis-Blériot, 63000 Clermont-Ferrand, France; celine.olmiere@theaopeninnovation.com; 5 Centre Anticancéreux Georges François Leclerc Center, 21000 Dijon, France

**Keywords:** AMD, angiogenesis, ocular diseases, VEGF, VEGF-receptor, omega-3 fatty acids, resveratrol

## Abstract

Age-related macular degeneration (AMD) is one of the main causes of deterioration in vision in adults aged 55 and older. In spite of therapies, the progression of the disease is often observed without reverse vision quality. In the present study, we explored whether, in undifferentiated ARPE-19 retinal cells, a disruption of the VEGF receptors (VEGF-R)/caveolin-1 (Cav-1)/protein kinases pathway could be a target for counteracting VEGF secretion. We highlight that Resvega^®^, a combination of omega-3 fatty acids with an antioxidant, resveratrol, inhibits VEGF-A secretion in vitro by disrupting the dissociation of the VEGF-R2/Cav-1 complex into rafts and subsequently preventing MAPK activation. Moreover, DNA ChIP analysis reveals that this combination prevents the interaction between AP-1 and *vegf-a* and *vegf-r2* gene promoters. By these pathways, Resvega could present a potential interest as nutritional complementation against AMD.

## 1. Introduction

Among the degenerative pathologies linked to age, eye diseases represent a significant part, and in particular age-related macular degeneration (AMD), which is one of the main causes of deterioration in vision in adults aged 55 and older in developed countries with an obvious negative impact on quality of life [1]. This disease is characterized by damage to the macula, the central area of the retina, which allows for fine or central vision. AMD is characterized by different key steps such as oxidative damage, lipofuscin accumulation, and impaired activity or function of the retinal pigmented epithelium (RPE). Subsequently, increased cell death is observed along with inflammation, which is activated by the accumulation of extracellular and intracellular debris. Finally, there is the development of atrophy (“dry AMD”) or of so-called new vessels in the macular region that seem to play a very important role in the complications associated with “wet AMD”. The first warning signs—deformation of straight lines, abrupt decrease in visual acuity or contrasts, dark central spot—should lead to prompt consultation at an ophthalmologist. It is usually classed into one of two forms. For the atrophic form, only nutritional supplementation is currently given since no therapies have been shown to be effective. The aim of the non-exudative AMD treatment is to delay the loss of visual function. The second form is a wet or “exudative” or “neovascular” form. The abnormal vessels are fragile and allow the serum to diffuse, which can cause serious detachment and lead to hemorrhage. The disease progression will depend on the type and location of these abnormal vessels, the possible occurrence of retinal uplifts, bleeding, and the response to treatment. It is crucial to detect the first signs of exudative macular degeneration because the earlier the diagnosis is made, the more effective the treatment. As AMD involves several factors such as oxidative stress, inflammation, and angiogenesis, antioxidants as vitamin E or fatty acids, especially poly-unsaturated acids (PUFA), could be used as supplementation to protect against AMD through their antioxidant power. In this way, the Age-Related Eye Disease Study 1 (AREDS-1), a multicenter, randomized controlled clinical trial, demonstrated that oral nutritional supplementation of a combination of vitamin C, vitamin E, β-carotene, zinc oxide, and cupric oxide in patients with intermediate or advanced AMD in one eye had a 25% relative risk reduction over 5 years of developing advanced AMD. The risk of vision loss of three or more lines was reduced by 19% with this supplementation [2]. Moreover, a French study (NAT1, Nutritional AMD Treatment 1) showed that lesions due to AMD were stabilized in patients supplemented with a PUFA, the docosahexaenoic acid (DHA) [3]. Alongside a preventive supplementation with nutraceuticals, therapies used for wet AMD aim to inhibit abnormal growth of blood vessels with laser photocoagulation or vascular endothelial growth factor (VEGF) inhibitors that are injected into the eye; however, some side effects and progression of AMD have been observed, and anti-VEGF-A therapies do not reverse vision quality.

Faced with this major public health problem, numerous studies and pharmaceutical companies have attempted to develop other inhibitors of VEGF, which is the key factor controlling neoangiogenesis analogous to what is well known in models of tumor angiogenesis. Indeed, the molecular mechanisms by which VEGF induces activation of kinase cascades from the different isoforms of its receptor, VEGF-R, are well described. More specifically, some authors have been able to show the importance of constitutive proteins from lipid rafts, such as the caveolin-1 (Cav-1) protein, in the early activation phase induced by VEGF in cancer models [4]. The binding of the latter to the extracellular domain of its receptor induces a signaling pathway initiated in low-density caveolar membrane domains and requires the dissociation of the receptor to Cav-1. The latter acts as a negative regulator of VEG-R2 activity under basal conditions and as a substrate that is tyrosine-phosphorylated under activating conditions [4].

Herein, we sought to fill the gap in the literature concerning the link between retinal cells and the disruption of the fine molecular mechanism involved in VEGF cascades. To this end, we explored in retinal cells mimicking the AMD phenotype whether a disruption in the sequence of VEGF-R/Cav-1/protein kinase activation could be a potential target for counteracting VEGF secretion in retinal cells and, subsequently, AMD progression. We use a specific nutraceutical, Resvega^®^, which in comparison to the AREDS formulations, Resvega^®^ (RSG) has resveratrol (RSV) as an additional component [5,6]. In this study, we highlight in retinal cells with an AMD phenotype that VEGF-R2 is dissociated to Cav-1 to activate the Raf–mitogen-activated protein kinase (MAPK)–extracellular signal-regulated kinases 1/2 (ERK1/2) oncogenic signaling pathway. The nutraceutical composed of both omega-3 fatty acids (ω3) and resveratrol (RSV), a polyphenol of grapevines, strongly favors VEGF-R2/Cav-1 association and decreases the active phosphorylated form of Cav-1. These events lead to inhibition of the oncogenic signaling pathway and target the transcriptional activation of *vegf-r2* and *vegf* gene expression. Subsequently, protein expressions are altered, and the VEGF-VEGF-R2 loop is inhibited.

## 2. Results

### 2.1. Combination of Resveratrol/ω3 Fatty Acid Inhibits VEGF Pathway in ARPE-19 Cells

**Figure 1:**
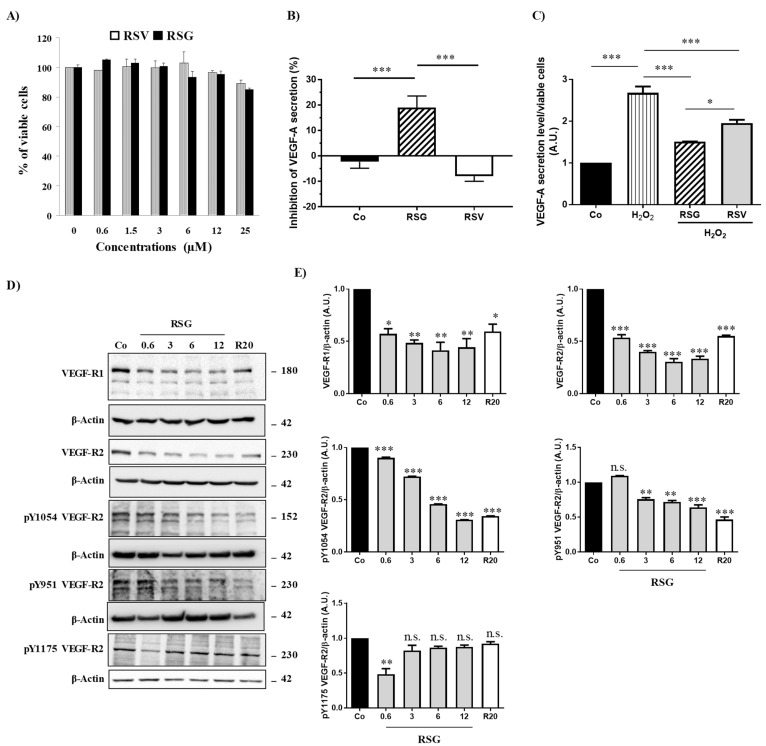
RSV/ω3 fatty acids combination disrupts VEGF-A secretion and inhibits phosphorylation of its receptor. (**A**) Determination of RSG and RSV effects on human retinal ARPE-19 cells. After treatment of ARPE-19 cells with increasing of RSG (0; 0.6; 3; 6; and 12 µM) or RSV (20 µM) concentrations at 37 °C for 24 h, the percentage of cell viability was determined by crystal violet assay. Results are expressed as a percentage of control (mean ± standard deviation of three independent experiments with *n =* 10). (**B**) ARPE-19 retina cells were treated for 24 h without serum with RSG (12 µM) or RSV (20 µM). VEGF-A secretion was measured in cell medium by ELISA. The data are mean ± S.D. of four independent experiments with *n* = 10. *p* values were determined by one-way ANOVA followed by Tukey’s multiple comparison test. * = *p* < 0.05; ** = *p* < 0.01; *** = *p* < 0.001. (**C**) ARPE-19 retina cells were treated without (Co) or with H_2_O_2_ (200 µM) for 24 h in combination or not with RSG (12 µM) or RSV (20 µM). As in (A) VEGF-A secretion was measured in cell medium by ELISA. The data are mean ± S.D. of four independent experiments with *n* = 10. *p* values were determined by one-way ANOVA followed by Tukey’s multiple comparison test. * = *p* < 0.05; ** = *p* < 0.01; *** = *p* < 0.001. (**D**) Immunoblot analysis of VEGF-R1, VEGF-R2, phospho Y1054 VEGF-R2, phospho Y951 VEGF-R2, phospho Y115 VEGF-R2 in ARPE-19 cell line after treatment without (Co) or with RSG (0.6, 3, 6, 12 µM) or with RSV (20 µM) for 24 h. β-actin was used as the loading control. (**E**) Densitometry quantification of western blots. Data are expressed as the mean fold induction ± SEM of three independent experiments. *p* values were determined by a one-way ANOVA followed by Tukey’s multiple comparison test. * *p* < 0.05, ** *p* < 0.01, *** *p* < 0.001 and n.s.: not significant.

The progression and complication of AMD result from excessive angiogenesis, where the vascular endothelial growth factor A (VEGF-A) plays a key role by contributing to the abnormal growth of blood vessels and an increase in vascular permeability that leads to loss of vision [7]. Due to the ability of RSV to limit production of VEGF in various models of cancer and retinal cells [8,9,10], we determined the ability of a nutraceutical, Resvega^®^ (RSG) containing both RSV and ω3 fatty acids (EPA: eicosapentaenoic acid, DHA: docosahexaenoic acid), to decrease the production of VEGF-A in ARPE-19, a retinal cell model spontaneously producing VEGF and mimicking the AMD phenotype [11]. For these experiments, we use undifferentiated ARPE-19 retinal cells mimicking cells affected by AMD [12] and that are widely used to test the effect of various molecules and anti-angiogenic compounds, including RSG and RSV [13,14]. Before evaluating VEGF secretion, we first assessed the toxicity of RSG and RSV on undifferentiated ARPE-19 retinal cells. In order to examine the effects of RSG, it was added to cells at concentrations that corresponded to RSV concentrations 0.6; 1.5; 3; 6; 12; 25 µM, since beyond this concentration, RSG has been shown to decrease metabolic activity (especially at 50 and 100 µM) [14]. It appears that neither RSV nor RSG has a significant impact on the cellular viability of human retinal cells ARPE-19 after 24 h of treatment with a range of increasing concentrations from 0 up to 25 µM (Figure 1A). The absence of toxicity of resveratrol at the concentrations used confirms our data published previously on this cellular model [15]. In view of these first results, RSG was therefore used at these non-toxic concentrations (0.6; 3; 6; 12 µM) during the 24 h of treatment in the ARPE-19 cell line, and RSV serves as a positive control for the concentration of 20 µM.

In basal conditions, it appeared that a 24 h treatment with RSV (20 µM) did not decrease VEGF-A secretion from these cells and even slightly increased it (Figure 1B). Very surprisingly, a combination of ω3/RSV at a very low dose of RSV (12 µM) showed a significant decrease in VEGF-A secretion from retinal ARPE-19 cells compared with the control or with RSV alone (Figure 1B). This inhibitory action induced by RSG is more important when retinal cells are previously pretreated with a well-known inducer of VEFG-A secretion such as H_2_O_2_. Indeed, when the retinal cells are subjected to continuous but non-toxic oxidative stress, there is an overproduction of VEGF-A by H_2_O_2_ (200 µM), which is almost tripled compared with the control (Figure 1C). However, when ARPE-19 cells are cotreated with RSG, the overproduction of VEGF-A is reduced by 43% compared with H_2_O_2_ treatment alone. In comparison with RSV that decreases modestly by 27%, the H_2_O_2_-induced VEGF-A secretion, RSG, appeared to be the most effective in reducing VEGF-A secretion (Figure 1C).

Secretion of VEGF by retinal cells results from an activation of the signaling pathway involving VEGF-specific tyrosine kinase receptors whose activation loop results from a phosphorylation cascade through the induction of successive kinases [16]. In order to evaluate the impact of the ω3 fatty acids/RSV combination on the activating phosphorylation pathway, we next investigated the influence of the different treatments on global protein phosphorylation in ARPE-19 cells. By using an antibody targeting the total phosphorylation sites, we showed in the same basal conditions that only RSG was able to decrease global protein phosphorylation compared with the control (Supplementary Figure S1A). As expected, when retinal cells were stimulated with a recombinant VEGF-A at 10 ng/mL, a high global phosphorylation level is observed in control cells. The VEGF-A increasing effect on phosphorylation level is lowered when cells are pre-treated for 24 h with RSG (Supplementary Figure S1B). This overall decrease in VEGF-induced phosphorylation suggests that the signaling pathway involving the activation of multiple kinase cascades may be affected. Indeed, in the canonic pathway, VEGF-A can bind to two distinct VEGF receptors (VEGF-R) with tyrosine kinase domains leading to homo- or hetero- dimer formation: VEGF-R1, also known as fms-like tyrosine kinase (Flt-1) and VEGF-R2, also known as fetal liver kinase (Flk-1/KDR) [17]. In retinal cells, VEGF binds to VEGF-R2, leading to the dimerization of the receptor and the activation of its intracellular domain through the phosphorylation of tyrosine residues. Once activated, the signaling cascade is triggered, allowing for the recruitment and activation of various factors leading to the transcriptional activation of the gene coding for VEGF and consequently to its production. We found that a treatment with a ω-3/RSV combination (RSG) for 24 h was able to significantly decrease in a concentration-dependent manner the expressions of VEGF-R1 and -R2 compared to the control (Figure 1D). More interestingly, the 6 µM RSG treatment induced a more significant decrease of VEGFR2 than RSV alone at a concentration of 20 µM (Figure 1D).

VEGF-R2 is the receptor most involved in the signaling cascade through the phosphorylation of its intracytoplasmic domain. Different tyrosine (Y) sites of VEGF-R2 are particularly important—Y1175, Y1054, and Y951—for kinase regulation and vascular permeability [18]. We investigated among these phosphorylation sites which ones could be more affected by the different treatments. We found that RSG drastically decreased VEGF-R2 activation by decreasing the phosphorylation of both Y1054 and Y951 tyrosyl residues with a very clear inhibition for Y1054, as compared with the control (Figure 1D,E). In the same manner, p-Y951 VEGF-R2 was significantly decreased through RSG treatment from 3 µM of ω-3/RSV combination (Figure 1D,E). The Y1175 phosphorylation site seems not to be affected by the different treatments.

### 2.2. VEGFR Signaling Pathway Is Disrupted via Lipid Rafts

**Figure 2:**
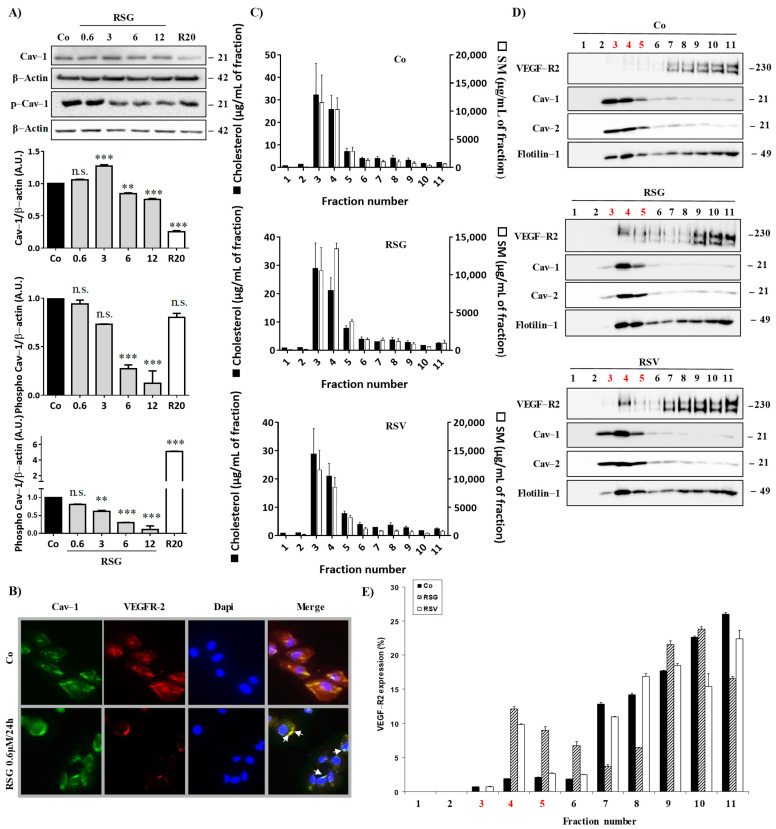
Disruption of VEGF-R2/Cav-1 dissociation into lipid rafts by RSV/ω3 fatty acids combination. (**A**) ARPE-19 retina cells were treated for 24 h by increasing concentrations of RSG (0.6, 3, 6, 12 µM) or with RSV (20 µM) for 24 h. A representative blot of Cav-1 and phospho-Cav-1 (p-Cav-1) is shown from three independent experiments. β-actin was used as a loading control. Densitometry quantification of western blotting is expressed as the mean fold induction ± SEM of three independent experiments. *p* values were determined by a one-way ANOVA followed by Tukey’s multiple comparison test. ** *p* < 0.01, *** *p* < 0.001 and n.s.: not significant. (**B**) ARPE-19 cells were left untreated (Co) or treated with 0.6 µM RSG for 24 h before fluorescence microscopy analysis of caveolin-1 (Cav-1) and VEGF-R2 coexpression. A representative image of three independent experiments is shown. (**C**) Lipid composition of ARPE-19 cell lysate fractions after RSG (12 µM) or RSV (20 µM) treatment for 24 h. Cholesterol (black bars) and sphingomyelin (white bars) were expressed in µg/mL of a fraction. Data are expressed as the mean fold induction ± SEM of three independent experiments. (**D**) The representative blot of the expression of indicated proteins in the above-defined fractions is shown from three independent experiments. (**E**) Densitometry quantification of western blotting. Data are expressed as the mean fold induction ± SEM of three independent experiments.

We next explored the mechanisms underlying the regulation of VEGF-R2 activity, which is poorly understood, especially in retinal cells. Several reports have suggested that VEGF-R2 may be localized in detergent-resistant membranes (DRM) known as lipid rafts, playing the role of a signaling platform to initiate a cascade of kinases in endothelial cells [4]. Then, VEGF-R2 is endocytosed within small invaginations (caveolae) mainly formed by caveolin, a constitutive protein of lipid rafts. Since caveolin-1 (Cav-1) is highly expressed in the eye [19] and is up-regulated during ocular neovascularization [20], we next explored the impact of the treatments with RSG and RSV on Cav-1 protein expression and its active phosphorylated form. Immunoblotting analysis revealed that RSG slightly decreases Cav-1 protein expression and, more importantly, its phosphorylated form in a concentration-dependent manner (Figure 2A). To better characterize the link with lipid rafts, by using both anti-VEGF-R2 and anti-Cav-1 Abs, we chose to use the 0.6 μM concentration for RSG, because the latter is the lowest concentration allowing both a decrease in the VEGF-R2 while maintaining the expression of Cav-1 in order to be able to observe their potential localization. We first confirmed by fluorescence microscopy in retinal ARPE-19 cells a strong decrease in VEGF-R2 expression, and we also saw a well-localized fluorescence (Figure 2B). However, very interestingly, we found that VEGF-R2 colocalized with the raft-associated protein Cav-1 at the surface of RSG-treated retinal cells, as shown by a few yellow spots on the overlay, which is not observed in control cells (Figure 2B).

These points are particularly important since it has been previously shown in aortic bovine endothelial cells (BAEC) that phospho-Cav-1 functions as a scaffolding protein for the VEGF-mediated signaling pathway [21], and the egress of VEGF-R2 from caveolae/lipid rafts is concurrent with the tyrosine phosphorylation of caveolin-1 [4]. Thus, these two events, decreasing the active form of Cav-1 and maintaining VEGF-R2 with Cav-1-associated in lipid rafts, could contribute to the ability of RSG to maintain the inactive form of VEGF-R2 associated with Cav-1 in lipid rafts. To better characterize the involvement of lipid rafts in this VEGF-R signaling pathway in retinal cells, we isolated lipid rafts from ARPE-19 treated with the different compounds. Lysates of ARPE-19 exposed or not for 24 h to RSG or RSV were fractionated on a sucrose gradient, and the lipid content of each fraction was determined by HPLC-coupled mass spectrometry to identify sphingomyelin- and cholesterol-enriched fractions corresponding to lipid rafts (Figure 2C). Further, immunoblotting of flotillin and the two isoforms of caveolin, Cav-1 and Cav-2, confirmed the lipid raft enrichment in fractions 3, 4, and 5 (Figure 2D). Immunoblot analysis of receptors to VEGF indicated that VEGF-R2 was almost undetectable in lipid raft fractions (3, 4, and 5) in control cells, as shown by the western blot and qualitative analysis of three independent experiments, while rafts marker proteins were perfectly localized in these fractions rich in cholesterol and sphingomyelin (Figure 2C,D). Conversely, RSG and RSV treatments strongly relocalized VEGF-R2 into fractions 3, 4, and 5, corresponding to lipid rafts, especially in fraction 4 where Cav-1 was mainly detected (Figure 2D,E). Thus, these results reinforced microscopy observations in which RSG colocalized with Cav-1 into lipid rafts (Figure 2B). Activation of the VEGF-R2 signaling pathway involves the dissociation of VEGF-R2 to Cav-1, leading to Cav-1 phosphorylation, which induces a kinase cascade as shown previously in bovine BAEC cells [4]. Thus, by being delocalized in lipid rafts at a distance from Cav-1, the VEGF-R2 signaling pathway could be reduced or even inhibited by treatment with RSG. In order to verify this hypothesis, we analyzed if RSG could modulate these phosphorylation cascades.

### 2.3. ω-3. Fatty Acids/RSV Combination-Inhibited VEGF-R2 Activation Is Associated with Disruption of MAPK Pathway Activation

**Figure 3:**
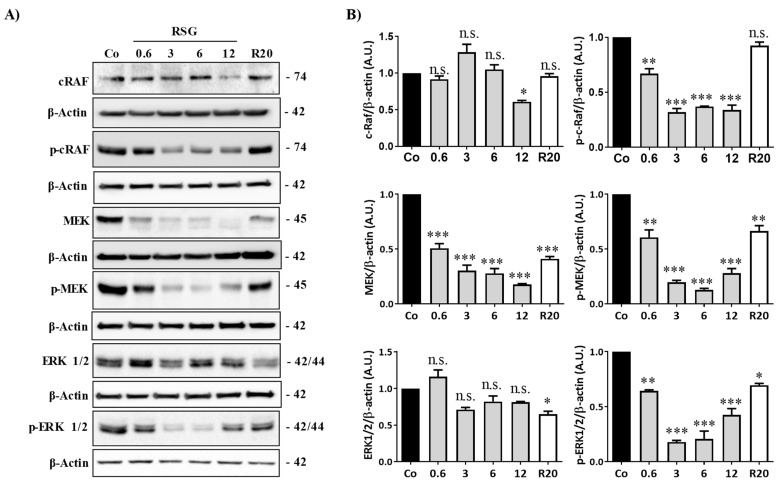
RSG affects the Raf-MAP kinases pathway in retinal cells. (**A**) Immunoblot analysis of c-Raf, phospho c-Raf (p-Raf), MEK, phospho MEK (p-MEK), ERK 1/2, phospho-ERK 1/2 (p ERK 1/2) in RSG-treated ARPE-19 cells with increasing concentration (0.6, 3, 6, 12 µM) or with RSV (20 µM) for 24 h. β-actin was used as a loading control. (**B**) Densitometry quantification of western blotting. Data are expressed as the mean fold induction ± SEM of three independent experiments. P values were determined by a one-way ANOVA followed by Tukey’s multiple comparison test. * *p* < 0.05, ** *p* < 0.01 and *** *p* < 0.001, n.s.: not significant.

When tyrosine kinase domains are autophosphorylated in their cytoplasmic domains, the downstream signal can be triggered to initiate and activate the signaling pathway [16]. The Raf-mitogen-activated protein kinase (MAPK)-extracellular signal-regulated kinases (ERK1/2) represents an important oncogenic signaling pathway to target the transcriptional activation of *vegf-r2* and *vegf* gene expression and, subsequently, protein expression [22]. We observed that RSG strongly decreased in a concentration-dependent manner the different protein kinases involved and their phosphorylated forms, as shown for Raf, MEK, and ERK1/2 protein expression (Figure 3A,B). Once again, as compared with RSV alone, RSG had a better effect on inhibiting this sequence of kinase activation.

Altogether, these first data demonstrate that RSG containing the ω-3 fatty acids/RSV combination act in synergy to alter the signaling pathway of VEGF-R2 in human retinal cells mimicking the AMD phenotype.

### 2.4. ω-3. Fatty Acids/RSV Combination Affects c-Jun/c-Fos Signaling Pathways and Their Relocalization into the Nucleus

**Figure 4:**
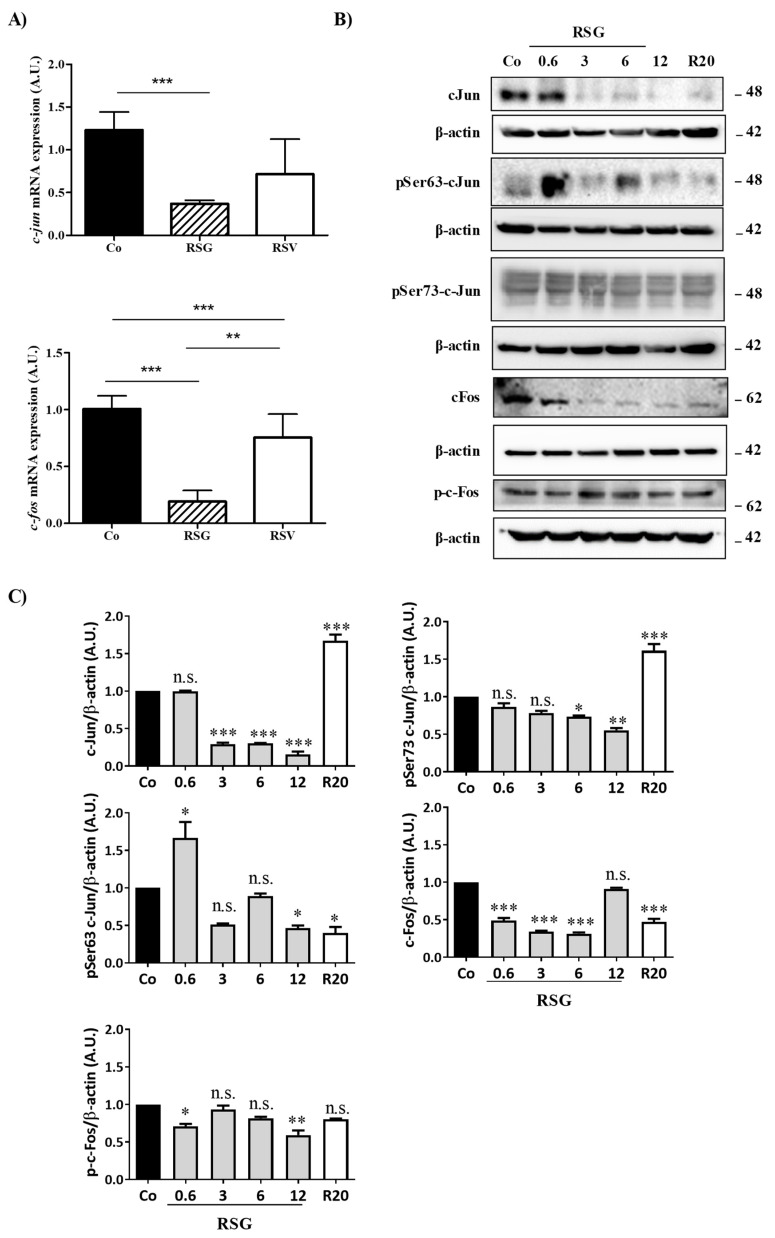
RSG down-regulates nuclear transcriptional factors c-Jun and c-Fos and their phosphorylated forms. (**A**) RT-qPCR mRNA expression analysis of *c-jun* and *c-fos*. Data represent three independent experiments. Data are expressed as the mean fold induction ± SEM of three independent experiments. *p* values were determined by a one-way ANOVA followed by Tukey’s multiple comparison test. * *p* < 0.05, ** *p* < 0.01, *** *p* < 0.001 and n.s.: not significant. (**B**) Immunoblot analysis of c-Jun, phospho Ser63 c-Jun (pSer63 c-Jun), phospho Ser73 c-Jun (pSer73 c-Jun), c-Fos, phospho-c-Fos (p-c-Fos) in RSG-treated ARPE-19 cells with increasing concentration (0.6, 3, 6, 12 µM) or with RSV (20 µM) for 24 h. β-actin was used as the loading control. (**C**) Densitometry quantification of western blotting. Data are expressed as the mean fold induction ± SEM of three independent experiments. *p* values were determined by a one-way ANOVA followed by Tukey’s multiple comparison test. * *p* < 0.05, ** *p* < 0.01 and *** *p* < 0.001.

Disruption of the intracellular signaling pathway involved in VEGF-R2 by RSG, especially the members of the classic MAPK cascades (MEK, ERK1/2), could affect the ultimate nuclear factors such as c-Jun and c-Fos. These transcriptional factors can form a homodimerization between c-Jun or a heterodimerization between c-Jun and c-Fos, leading to the formation of the transcription factor activator protein 1 (AP-1) complex [23]. Transcriptional activity of AP-1 complexes is primarily mediated by induction of their expression and regulated by post-translational modification, particularly their phosphorylation by MAP kinase family members such as ERK 1/2. Considering the most important role played by this transcriptional factor, we next assessed whether the previous modulation of MAPK by RSG could have an impact on c-Jun and c-Fos protein and gene expression and determined the potential key role of AP-1 in RSG actions. To this end, we treated retinal cells with RSG and RSV for 24 h. We observed a strong decrease in both *c-jun* and *c-fos* mRNA expression with RSG, which was more significant after 24 h of treatment (Figure 4A). This decrease in gene expression was associated with a strong decrease in c-Jun and c-Fos protein expression in a concentration-dependent manner with RSG, which was also observed with 24 h of treatment with RSV (Figure 4B,C). Post-translational events are important for the increase in transcriptional activity of c-Jun of c-Fos, such as increases of phosphorylation on their serine sites in their transactivation domain [24,25]. Very surprisingly, no treatments modulated the phosphorylation of c-Fos; however, RSG strongly disrupted phosphorylation of c-Jun (Figure 4B,C). Indeed, using specific Abs against serine residues of c-Jun, we observed a decrease in phospho Ser63-c-Jun with RSG and RSV and a slight modulation of phospho Ser73-c-Jun at 12 µM of RSG (Figure 4B,C).

Phosphorylation at Ser 63 and 73 is crucial for the role of c-Jun. Indeed, the transcriptional activity of c-Jun is increased by phosphorylation of these two serines in the transactivation domain that are mediated by MAP kinases [24,25]. To act as transcriptional factors and to form the homo- or heterodimer AP-1, c-Jun and its phosphorylated forms must be translocated into the nucleus to response elements of target genes.

**Figure 5:**
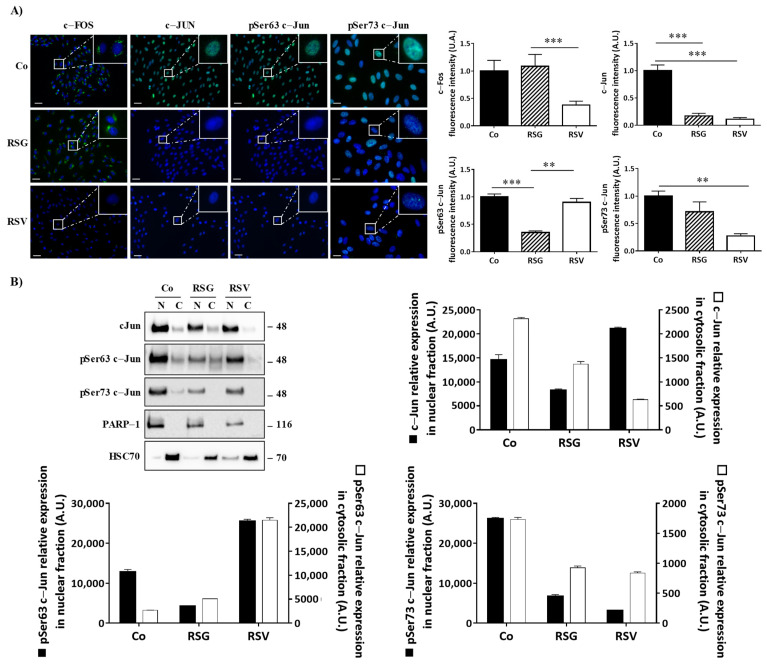
RSG inhibits the translocation of phosphorylated c-Jun forms into the nucleus of ARPE-19 cells. (**A**) Immunofluorescence analysis of expression and localization of c-Fos, c-Jun, and phospho Ser63 c-Jun, phospho Ser 73 c-Jun into untreated (Co) or treated ARPE-19 retina cells with RSG (12 µM) or with RSV (20 µM) for 24 h. Left panel, representative images of specific proteins expression (green fluorescence) counterstaining with Hoechst 33342 (nuclei, blue). Right panel quantification of a specific protein on merged pictures (300 cells per line) with Image J software. *p* values were determined by a one-way ANOVA followed by Tukey’s multiple comparison test. ** *p* < 0.01, *** *p* < 0.001 and n.s.: not significant. (**B**) Representative blot of c-Jun, phospho Ser63 c-Jun (pSer63 c-Jun), phospho Ser73 c-Jun (pSer73 c-Jun) protein expression in RSG (12 µM)-treated ARPE-19 cells or with RSV (20 µM) for 24 h. Nuclear fractions (N) and cytosolic (C) fractions are shown from three independent experiments. Densitometry quantification of western blotting. Data are expressed as the mean fold induction ± SEM of three independent experiments. *p* values were determined by a one-way ANOVA followed by Tukey’s multiple comparison test. ** *p* < 0.01 and *** *p* < 0.001.

Fluorescence microscopy analysis confirmed the decrease in the proteins of interest but showed that RSG abolished the migration of the transcriptional factors, c-Jun, and its phosphorylated forms Ser 63 and Ser73, into the nucleus (Figure 5A). Indeed, RSG halved the c-Jun protein amount compared with the control and the other treatments (Figure 5A).

To assess this relevant property of RSG in preventing the relocalization of nuclear factors into the nucleus, we performed immunoblotting from a nucleus/cytoplasm extraction where poly(ADP-ribose) polymerase-1 (PARP-1) and heat shock cognate (HSC70) proteins are used as controls for the nuclear and cytosolic fractions, respectively. While c-Jun and its active phosphorylated forms are mainly in the nucleus, as demonstrated in the control, RSG strongly decreases the amount of c-Jun protein in these nuclear fractions compared with the control by 44% (Figure 5B). In a similar manner for its phosphorylated forms, phospho-Ser63-c-Jun both strongly decreased in the nucleus fractions (RSG −67%) and strongly increased in the cytosolic fractions (Figure 5B). Surprisingly, RSV increased phospho-Ser63-c-Jun both in nuclear and cytosolic fractions compared with the control. On the other hand, phospho-Ser73-c-Jun also clearly decreased through RSG treatment in nuclear fractions compared with the control by 75% as well as through RSV by 80%, respectively (Figure 5B).

Collectively, these results indicate that a functional VEGF-R2 allows for the activation of a cascade of kinases, leading to the migration into the nucleus of the nuclear factors necessary for the AP-1 complex formation, which could play a role as a transcriptional activator of target genes. Conversely, treatment with a nutraceutical such as RSG keeps the VEGF-R2 complex in an inactive form and inactivates the Raf-MEK-ERK pathway, but also abolishes the translocation of c-Jun in the nucleus.

### 2.5. ω-3. Fatty Acids/RSV Combination Antagonizes AP-1 Binding Sites on VEGF and VEGF-r2 Promoter Genes and Their Transcription

**Figure 6:**
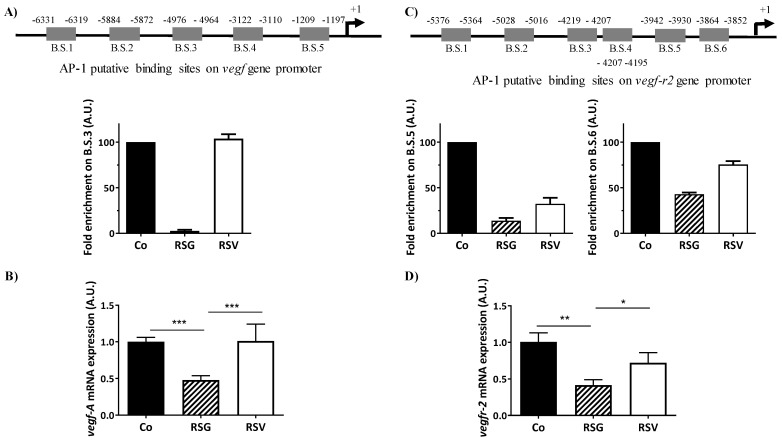
RSG inhibits *vegf-a* and *vegf-r2* mRNA expressions through a disruption of the AP-1 binding sites. ARPE-19 cells were untreated (Co) or treated with RSG (12 µM) or with RSV (20 µM) for 24 h. (**A**) Upper panel, analysis on the putative binding sites of AP-1 on the *vegf-a* gene promoter. Down panel, ChIP analysis of the interaction between AP-1 and the *vegf* promoter in ARPE-19 cells. Analysis of the putative B.S.3 for AP-1 on the *vegf* promoter (−4976 −4964) is shown. For the putative B.S.1, B.S.2, B.S.4, and B.S.5, any amplification was obtained. (**B**) RT-qPCR mRNA expression analysis of *vegf-a*. Data represent three independent experiments. Data are expressed as the mean fold induction ± SEM of three independent experiments. *p* values were determined by a one-way ANOVA followed by Tukey’s multiple comparison test. * *p* < 0.05, ** *p* < 0.01 and *** *p* < 0.001. (**C**) Upper panel, analysis of the putative binding sites of AP-1 on the *vegf-r2* gene promoter. Down panel, ChIP analysis of the interaction between AP-1 and the *vegf-r2* promoter in ARPE-19 cells. Analysis of the putative B.S.5 and B.S.6 for AP-1 on the *vegf-r2* promoter (−3942, −3930, and −3864, −3852, respectively) is shown. For the putative B.S.1, B.S.2, B.S.3, and B.S.4, any amplification was obtained. (**D**) RT-qPCR mRNA expression analysis of *vegf-r2*. Data represent three independent experiments. Data are expressed as the mean fold induction ± SEM of three independent experiments. *p* values were determined by a one-way ANOVA followed by Tukey’s multiple comparison test. * *p* < 0.05, ** *p* < 0.01 and *** *p* < 0.001.

Activation of the Ras-MEK-ERK signaling pathway is known to up-regulate VEGF expression and also its receptor, VEGF-R2, in various models, particularly in cancer models, to promote angiogenesis [26]. A disruption of the final elements such as translocation of AP-1 into the nucleus should alter its transcriptional activity on the promoter of *vegf* and *vegf-r2* genes and subsequently must strongly affect the VEGF/VEGF-R2 autocrine feed-forward loop triggering angiogenesis. Alteration of c-Fos and especially c-Jun and its phosphorylated forms by RSG led us to explore whether this ω-3 fatty acids/RSV combination could antagonize the positive effect of the AP-1 transactivation factor on the promoters of the *vegf* and *vegf-r2* genes. Accordingly, AP-1 chromatin immunoprecipitation (ChIP) assay on five putative binding sites of the *vegf* promoter gene (Figure 6A, upper panel) using nuclear extracts of human retinal ARPE-19 cells treated or not treated with nutraceuticals RSG and RSV for 24 h showed that AP-1 on the *vegf* promoter (binding site 3 (B.S.3)) is dramatically impeded by RSG compared with the control or with RSV (no enrichment was observed on B.1; B.S.2; B.S. 4; B.S.5) (Figure 6A). This antagonization of AP-1 to bind and to activate the transcription of the *vegf* promoter gene was subsequently associated with a decrease in *vegf* mRNA levels after treatment with RSG (Figure 6B). The gene of the VEGF-R2 protein possesses in its proximal promoter region multiple AP-1 binding sites that could be involved in the nutraceutical action (Figure 6C, upper panel). As previously, the AP-1 ChIP assay on the six putative binding sites on the *vegf-r2* promoter gene showed that AP-1 on the *vegf-r2* promoter (binding sites 5 (B.S.5) and 6 (B.S.6)) is affected by RSG, with a stronger effect on (B.S.5) than (B.S.6) compared with the control (no enrichment was observed on B.1; B.S.2; B.S.3; B.S.4) (Figure 6C). RSV also seemed to disrupt and antagonize AP-1 binding sites (Figure 6C). In a similar manner to the *vegf* gene expression, this action induced by RSG and RSV led to a strong decrease in the *vegf-r2* mRNA levels (Figure 6D).

Together, these data highlight a potential role of RSG as a nutraceutical composed of polyphenols and ω-3 fatty acids to counteract the progression of AMD through a pleiotropic action in the retina by targeting key regulators of neoangiogenesis in retina cells.

## 3. Discussion

**Figure 7:**
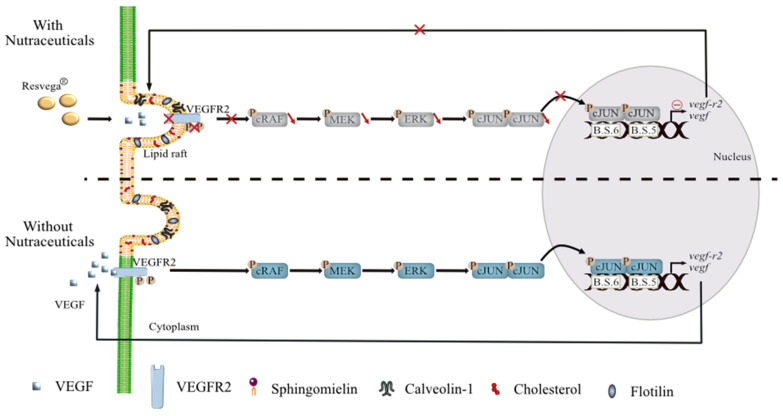
Suggested model to counteract VEGF-A secretion in retinal cells with AMD phenotype by nutraceuticals. Contrary to the physiopathological mechanism observed in retinal cells mimicking AMD, nutraceutical with resveratrol/ω3 fatty acids combination promotes (1) VEGF-R2 relocalization into lipid rafts and its binding with Cav-1, a constitutive protein of lipid rafts. This VEGF-R2-Cav-1 association, which acts as a negative regulator of the VEGF-A signaling cascade, prevents (2) the activation of MAP kinase pathway and subsequently decreases (3) phosphorylation of transcriptional nuclear factors such as c-Jun. Reduction of phospho-c-Jun level is associated with a (4) disruption of c-Jun into the nucleus. Furthermore, nutraceuticals also seemed to disrupt and antagonize AP-1 binding sites on the promoters of *vegf* and *vegfr* genes and contribute to the decrease in their mRNA and protein levels.

Despite an increasing number of reports highlighting the key role of VEGF-A in the development of blood vessels in tumors and other tissues undergoing abnormal angiogenesis [27,28], and the development of new anti-VEGF antibodies to counteract neovascularization, especially in advanced cancers or in ocular diseases (i.e., ranibizumab, bevacizumab, aflibercept, etc.) [29], side effects and the progression of AMD continue to be observed and lead to treatment failures. Furthermore, some studies have suggested a role for caveolin-1 (Cav-1), a protein of lipid rafts, in the regulation of the VEGF-R pathway in prostate cancer and endothelial cells [4,30]. Indeed, Cav-1 regulates VEGF-stimulated VEGF-R2 autophosphorylation and the activation of downstream angiogenic signaling, possibly through compartmentalization of specific signaling molecules. Caveolae and lipid rafts act as platforms for the negative modulation of VEGF signal transduction cascades in various cancer models (Figure 7) [4,31]. However, no direct links between VEGF-R2, Cav-1, their localization into lipid rafts, and the full mechanism induced in ocular degenerative diseases such as AMD have been provided so far. Herein, for the first time, through the use of potential anti-angiogenic nutraceuticals, we provide a mechanism by which a ω-3 fatty acids/RSV combination (RSG) can counteract VEGF secretion through modulation of the link between VEGF-R2 and Cav-1 into lipid rafts and the repercussions on the downstream signaling cascade.

The pathological process of AMD leads to progressive destruction of the neurosensory macular area, involving the RPE, Bruch’s membrane, and the choroid. Different stages of the disease have been described with the manifestation of retinal abnormalities and the appearance of drusen (extracellular deposit of lipid, cellular debris, and protein) near the fovea, while late stages present with geographic atrophy or CNV. Angiogenesis, namely, the development of new blood vessels from pre-existing vasculature, plays a crucial role in neovascular AMD where pro-angiogenic VEGF-A had been shown to be involved in the development of CNV [32]. Based on their use in treatments for various cancers with metastasis, VEGF inhibitors have revolutionized the care of vasoproliferative ophthalmologic disease, but some side effects are observed. Subsequently, new alternatives and compounds have been researched to improve the management of this serious disease. For many years, various studies have sought alternatives or therapeutic supplements in order to prevent the occurrence of the disease or its progression. In this way, the Age-Related Eye Disease Study 1 (AREDS-1), a multicenter, randomized controlled clinical trial, demonstrated that oral nutritional supplementation of a combination of vitamin C (500 mg), vitamin E (400 UI), β-carotene (15 mg), zinc oxide (80 mg), and cupric oxide (2 mg) in patients with intermediate or advanced AMD in one eye had a 25% relative risk reduction of developing advanced AMD in the eye over 5 years. The risk of vision loss of three or more lines was reduced by 19% with this treatment. Moreover, several epidemiological studies demonstrated that carotenoid intake reduced the risk of advanced AMD and that a lutein- and zeaxanthin-based diet might protect against intermediate AMD in female patients. Thus, the objective of the second AREDS (AREDS-2) was to determine whether the addition of lutein/zeaxanthin and omega-3 fatty acids (docosahexaenoic (DHA) and eicosapentaenoic acids (EPA)) would further reduce progression to late-stage AMD. The addition of lutein + zeaxanthin, DHA + EPA, or lutein + zeaxanthin and DHA + EPA to the complete AREDS formulation did not further reduce the risk of progression to advanced AMD [2,5]. Moreover, because of the potential increased incidence of lung cancer in former smokers, lutein + zeaxanthin could be an appropriate carotenoid substitute in the AREDS formulation [5]. Nevertheless, other studies have shown that long-chain poly-unsaturated fatty acids could protect against AMD [3]. Indeed, a meta-analysis suggests that ω-3 fatty acids such as DHA, which are essential dietary compounds found in fish such as salmon or tuna, could reduce the risk of both early and late AMD [33]. In the same manner, other natural compounds, such as polyphenols, could act on the different molecular steps of AMD through their pleiotropic actions such as their antioxidant, anti-inflammatory, and anti-angiogenic properties [34,35,36]. These phytomolecules have cellular targets similar to those of the new drugs developed by pharmaceutical companies, and more than 1600 patents are currently reported concerning flavonoids and 3000 patents concerning polyphenols. One of the best known is the polyphenol resveratrol (RSV), which is a *trans*-3,4′,5-trihydroxystilbene and seems to be of great interest for the prevention of various pathologies. Indeed, RSV presents a myriad of health benefits and acts at multiple levels such as cellular signaling, enzymatic pathways, apoptosis, and gene expression to prevent or to fight coronary heart damage, cancers, or degenerative diseases (see for review [37,38,39]). It is well described that RSV can reduce cancer progression through an anti-angiogenic action [40]. This last property involved inhibition of VEGF at gene and protein expression levels through an NFκB-mediated mechanism, as we and others have previously shown in various cancer types such as melanoma or colon cancers [41,42]. More specifically, we have shown in retinal cells that RSV was able to reduce VEGF secretion induced by oxysterols and subsequently to counteract their toxic effects [15]. Recently, a clinical trial realized in the United States in octogenarian patients has shown that oral administration of a polyphenol combination including resveratrol and quercetin reduces neovascularization, and the authors saw objective retinal and visual restoration, similar to anti-VEGF therapy [43,44]. Based on these findings, we compared the action of resveratrol alone (RSV) with the ω-3 fatty acids/RSV combination (RSG) on human retinal cells mimicking the AMD phenotype with a VEGF secretion. Interestingly, the ω-3 fatty acids/RSV combination showed a synergic anti-angiogenic action compared with the two drugs alone, particularly when VEGF-inducers stimulated retinal cells. These results are in accordance with a previous study showing that RSG inhibited retinal endothelial tube formation. In fact, a concentration range of Resvega (25, 50, and 100 µM) reduced the ability of endothelial cells obtained from a human umbilical vein (HUVEC) and of monkey retinal endothelial cells (RF/6A) to form networks on matrigel [45]). Moreover, we demonstrate that RSG strongly decreased both VEGF-R2 activation through its tyrosine phosphorylation but also decreased Cav-1 phosphorylation. Cav-1 is expressed in ocular types, including vascular cells, RPE, and the lens epithelium (Figure 7) [46,47]. In the eye, Cav-1 plays an essential role in modulating angiogenic signaling where VEGF-R2 is localized into caveolae. Morais et al. showed that Cav-1 expression promotes VEGF-induced corneal angiogenesis [48]. Very interestingly, the ω-3 fatty acids/RSV combination (RSG), by decreasing the phosphorylation of Cav-1 into the residue Y14, altered the main function of phospho-Cav-1 that acts as a scaffolding protein for VEGF-mediated signaling by serving as a docking site for phospho-tyrosine-binding molecules [30]. Moreover, we observed that the ω-3 fatty acids/RSV combination reinforced the association between VEGF-R2 and Cav-1 into lipid rafts and subsequently disturbed the autophosphorylation of VEGF-R2 and activation of the downstream signaling cascade. According to the central role of the Raf-MAPK-ERK oncogenic signaling pathway in the control of transcription of VEGF-R2 and VEGF expression, we observed an important property of RSG that was able to inhibit the transcription of these genes (Figure 7). Indeed, we highlight that RSG was able to alter nuclear relocalization into the nucleus of nuclear transcriptional factors such as c-Jun and its phosphorylated form. This effect was reinforced by the fact that RSG was able to prevent the binding of the AP-1 complex on the response elements controlling gene transcription of *vegf-r2* and *vegf*. Subsequently, protein and gene expressions were reduced by the ω-3 fatty acids/RSV combination.

## 4. Materials and Methods

### 4.1. Cell Cultured and Viability Assays

The human retinal pigmented epithelial cell line ARPE-19, purchased from the American Type Culture Collection (Manassas, VA, USA), was maintained in Dulbecco’s Modified Eagle’s F12 medium (DMEM/F12) supplemented with 10% fetal bovine serum (Dutscher, Brumath, France), 1% penicillin/streptomycin in a humidified atmosphere of 5% CO_2_ at 37 °C. These undifferentiated ARPE-19 cells are spontaneously arising human RPE (retinal pigment epithelium) cell lines with normal karyology, which form polarized epithelial monolayers [49]. ARPE-19 has structural and functional properties characteristic of RPE cells in vivo. They both express some markers of RPE cells such as rpe65 and rlbp1 and various genes involved in the production of VEGF (i.e., rpe65, rlbp1, vegf, kdr, serpinf 1, pnp1). Due to these properties, ARPE-19 cells are considered as retinal cells, mimicking the AMD phenotype. Cells were seeded and grown to a sub-confluence of 60–70% in normoxia. After seeding for 24 h, the medium was removed, and the cells were washed once with Hank’s Balanced Salt Solution (HBSS, Dutscher, Brumath, France) before re-incubating in DMEMF12 with 1% FBS and 1% penicillin/streptomycin. The following day, cells were treated with DMSO, Resvega^®^ (Laboratoires Théa, Clermont-Ferrand, France), or Resveratrol (Sigma Aldrich, St. Quentin Fallavier, France). The viability assays were assessed by crystal violet staining (Sigma Aldrich, St. Quentin Fallavier, France). Briefly, retinal cells were seeded into 96-well plates and treated with increasing concentrations of RSG (Resvega^®^: Vitamin C 240 mg; E 30 mg; Zinc 12.5 mg; Cu 1 mg, EPA 380 mg; DHA 190 mg; Lutein 10 mg; Zeaxanthin 2 mg; *trans*-resveratrol 30 mg), and RSV for 0.5, 1, 2, 4, 6, 8, 24, 48, and 72 h. After 24 h of culture, at the end of the treatment, cells were washed with phosphate-buffered saline (PBS), fixed with ethanol for 10 min at 4°C, and then stained with a crystal violet solution (0.5% (*w/v*) crystal violet in 25% (*v/v*) methanol) for 15 min at room temperature. Cells were then gently rinsed with water, and absorbance was measured at 590 nm using a Biochrom Assays UVM 340 microplate reader, following extraction of the dye using an acetic acid 33% solution.

### 4.2. ELISA

Cell culture supernatants were assayed by ELISA for human VEGF-A (BMS277-2 Invitrogen, Waltham, MA, USA), according to the manufacturer’s protocol.

### 4.3. Western Blot Analysis

ARPE-19 cells washed with cold 1X phosphate-buffered saline (PBS Dutscher, Brumath, France) were either lysed in Boiling buffer (SDS sodium dodecyl sulfate 1%, orthovanadate 1 mM, Tris 10 mM (pH 7.4)) with protease inhibitors (Roche, Boulogne-Billancourt, France) for 30 min on ice. Then, the lysates were sonicated for 6/7 sec at 30% amplitude. Protein concentrations were measured using a BCA assay kit (Thermo Fisher Scientific, IllKirch-Graffenstaden, France). As for total protein, 25 to 60 μg were loaded onto 10% polyacrylamide gel. Proteins were resolved by SDS-PAGE and transferred to nitrocellulose membranes (Amersham, Les Ulis, France). Blots were then saturated in 5% milk (1 h at RT) before overnight incubation at 4 °C with specific primary antibodies (Supplementary Table S1). All primary antibodies were diluted at 1:1000 in 5% *w*/*v* non-fat milk or 5% BSA. Primary antibodies were detected using horseradish peroxidase (HRP)-conjugated appropriate secondary antibodies (Cell Signaling Technologies, IllKirch-Graffenstaden, France) followed by exposure to ECL (Santa Cruz Biotechnology, Nanterre, France). Signal was acquired with a ChemiDoc^TM^ XRS+ imaging system (Biorad, Marnes-la-coquette, France), and blots were analyzed with Image Lab^TM^ Software 5.1.2 (Biorad, Marnes-la-coquette, France).

### 4.4. Immunofluorescent Labelling and Staining of Cells

ARPE-19 cells cultured on coverslips were washed with cold PBS and fixed with 4% paraformaldehyde (PFA) for 10 min at room temperature, followed by permeabilization with cold methanol. The samples were blocked with 3% BSA 0.2% Saponin (47036, Sigma Aldrich) for 20 min at room temperature. Cells were incubated with primary antibody diluted 1:200 overnight at 4 °C. After washing with PBS, samples were incubated with secondary Alexa 488 or 568 coupled anti-rabbit (Life Technologies, Saint Aubin, France) diluted 1:1000 with BSA/Saponin buffer. After extensive washing, coverslips were mounted on a drop of Mounting Medium ProLong^®^ containing DAPI (Molecular Probes, ThermoFisher Scientific, Strasbourg, France) in the dark for 1 h. Slides were imaged using a CDD equipped upright microscope (Zeiss, Marly le Roi, France) and 63×, 1.4 NA objective.

### 4.5. Lipid Rafts Isolation and Biochemical Characterization

Retinal cells were grown in 175 cm^2^ dishes and treated for 24 h with or without treatments (RSG 12 µM, NUT 12 µM, and RSV 20 µM). Cells were washed with ice-cold PBS, harvested by scraping in 2 mL of ice-cold Tris-NaCl-EDTA buffer (TNE; 150 nM NaCl, 20 mM Tris (pH 7.4) and 1 mM EDTA) containing 1% (*w/v*) Lubrol, and vortexed. After 30 min of incubation on ice, cells were homogenized further by passing the lysate at least 25 times through a 21-gauge needle. Lysates were transferred to centrifuge tubes and mixed with 2 mL of 80% (*w*/*v*) sucrose in TNE. On top of this, 3.5 mL of 35% (*w*/*v*) and 3.5 mL of 5% (*w*/*v*) sucrose in TNE were successively loaded, resulting in a discontinuous gradient. All solutions contained a complete protease inhibition cocktail (Roche Applied Bioscience). The samples were centrifuged at 39,000 rpm for 20 h at 4 °C, and 1 mL fractions were collected from the top of the gradient. Then, 60 µL of each fraction was subjected to SDS-polyacrylamide gel electrophoresis and immunoblotted. Lipids were extracted and analyzed as described [50] in the indicated conditions. Expression profiles of both flotillin and caveolin-2, described as rafts markers, were analyzed in each fraction by western blotting.

### 4.6. Lipidomic Analysis of Lipid Rafts

For targeted analysis of cholesterol and sphingomyelins, 25 µL and 50 µL of rafts fractions were used by GCMS and LCMSMS, respectively, as previously described [51].

### 4.7. RNA Extraction and Quantitative PCR Analysis

Total cellular RNA was extracted with TRIzol^®^ RNA Isolation Reagent (Ambion). RNA (300 ng) was reverse transcribed into cDNA using M-MLV Reverse Transcriptase, random primers, and RNAseOUT inhibitor (Invitrogen). cDNA was quantified by real-time PCR with the Power SYBR Green PCR Master Mix (Applied Biosystems; Warrington, UK) on a 7500 Fast Real-Time PCR detection system (Applied Biosystems). Relative mRNA levels were determined by the ΔΔCt method and normalized to the expression levels of human or mouse *Actb.* Primer sequences used are listed in the following Table 1.

### 4.8. Nuclear and Cytoplasmic Fraction Isolation

The cells were lysed in buffer (NE-PER; Pierce Biotechnology, Rockford, IL, USA) supplemented with a protease inhibitor cocktail (Halt; Pierce Biotechnology, Rockford, IL, USA), according to the manufacturer’s protocol. Protein concentrations were determined (BCA Kit; Pierce Biotechnology) before being loaded onto polyacrylamide gels.

### 4.9. DNA ChIP Assay

Cells are prepared according to the truChIPTM Shearing Kit protocol (Covaris), collected, and rinsed using cold PBS 1 X. Cell pellets were fixed using buffer A and using 1% formaldehyde. After 10 min with stirring, we add buffer E then the cells are centrifuged (500 G, 5 min, room temperature). After two washes with PBS 1 X, buffer B is added to lyse plasma membranes (10 min with stirring at 4 °C). Then, cells are centrifuged, and the pellets resuspended in washing buffer C (10 min at 4 °C). The nuclei are recovered by centrifugation (1700 G, 5 min at 4 °C). The nuclear pellet is resuspended in the D3 buffer and transferred to milliTUBE AFA Fiber tubes (Covaris, Woburn, MA, USA). The samples are then ultrasound (3 sessions of 12 min). After this nuclear preparation, ChIP was conducted according to the manufacturer’s instruction: ChIP-IT^®^ Express Enzymatic (Active Motif, La Hulpe, Belgium) protocol. After dosing the chromatin, we carry out an incubation of 50 µg of chromatin with the magnetic beads, a supplied ChIP buffer, and the anti-VEGFR-2 (Cell Signaling, Danvers, MA, USA) or anti-VEGF (Cell signaling) antibodies overnight at 4 °C. The beads are then washed several times, and the chromatin is eluted using a buffer. The DNA obtained can be used immediately in PCR or stored at −20 °C. The sequences of the oligonucleotides used are described in Table 1.

### 4.10. Densitometry and Statistical Significance

The densitometry of blots was realized by the use of ImageJ software (National Institutes of Health). Unless indicated in the legends of figures, the reported values represent the means of triplicate from one representative experiment repeated three times +/− SD. Statistical significance was determined using the Mann–Whitney test at * *p* < 0.05, ** *p* < 0.01 or *** *p* < 0.001.

## 5. Conclusions

In this study, we showed that a nutraceutical enriched in omega-3 and in a polyphenol, resveratrol, could decrease VEGF-A secretion for retinal cells through a disruption of the MAP kinase pathway and the transcription of gene encoding to VEGF-R2 and VEGF proteins. Nonetheless, further investigations must be performed to demonstrate the potential use of Resvega as nutritional complementation against AMD, especially regarding whether these anti-angiogenic effects are confirmed in preclinical models of AMD.

## Figures and Tables

**Table 1:** Primers used for RT-qPCR analysis.

Gene	Forward Sequence	Reverse Sequence
***h-Beta-actin***	5′-TCCACCTTCCAGCAGATGTG-3′	5′-GCATTTGCGGTGGACGAT-3′
***h-vegf-a***	5′-ATCTTCAAGCCATCCTGTGTG-3′	5′-GAGGTTTGATCCGCATAATCTG-3′
***h-vegf-r1***	5′-GAAATCACCTACGTGCCGGA-3′	5′-ACGTTCAGATGGTGGCCAAT-3′
***h-vegf-r22***	5′-CCAGCAAAAGCAGGGAGTCTGT-3′	5′-TGTCTGTGTCATCGGAGTGATATCC-3′
***h-c-fos***	5′-TTATCTCCAGAAGAAGAAGAGAAAAGGAGAATC-3′	5′-AGGGCCAGCAGCGTGGGTGAGCTGAGCGAGTCA-3′
***h-c-jun***	5′-CAGCCAGGTCGGCAGTATAG-3′	5′-GGGACTCTGCCACTTGTCTC-3′
***h-c-jun #3 (vegf)***	5′-GAGCAGCGAAAGCGACAG-3′	5′-TGTCTGTCTGTCTGTCCGTCA-3′
***h-c-jun #5 (vegf-r2)***	5′-CACACCACACAGATGTGCAA-3′	5′-CCAATGCCAGTTAATTTCTGA-3′
***h-c-jun #6 (vegf-r2)***	5′-TCAGAAATTAACTGGCATTGG-3′	5′-AGACCCAGGGATATTCTGACA-3′

## Data Availability

The authors declare that all data supporting the findings of this study are available within the article.

## Supplementary Materials

The following are available online at https://www.mdpi.com/article/10.3390/ijms22126590/s1, Figure S1: RSG decreases expression of global phosphorylation, Table S1: Antibodies.
