# Supplementary material for: VEGF-R2/Caveolin-1 Pathway of Undifferentiated ARPE-19 Retina Cells: A Potential Target as Anti-VEGF-A Therapy in Wet AMD by Resvega, an Omega-3/Polyphenol Combination
Source: Int J Mol Sci. 2021 Jun 19;22(12):6590. doi: 10.3390/ijms22126590 (PMC8234996; doi:10.3390/ijms22126590)
Supplement: Supplementary file 1 [file ijms-22-06590-s001.zip › ijms-1250924-supplementary.pdf]

Supplementary Materials

VEGF-R2/Caveolin-1 Pathway of Undifferentiated ARPE-19 Retina Cells: A Potential Target as Anti-VEGF-A Therapy in Wet AMD by Resvega, an Omega-3/Polyphenol Combination

Flavie Courtaut [1,2,†], Alessandra Scagliarini [1,2,†], Virginie Aires [1,2,†], Clarisse Cornebise [1,2], Jean-Paul Pais de Barros [1,2,3], Céline Olmiere [4] and Dominique Delmas [1,2,5,*]

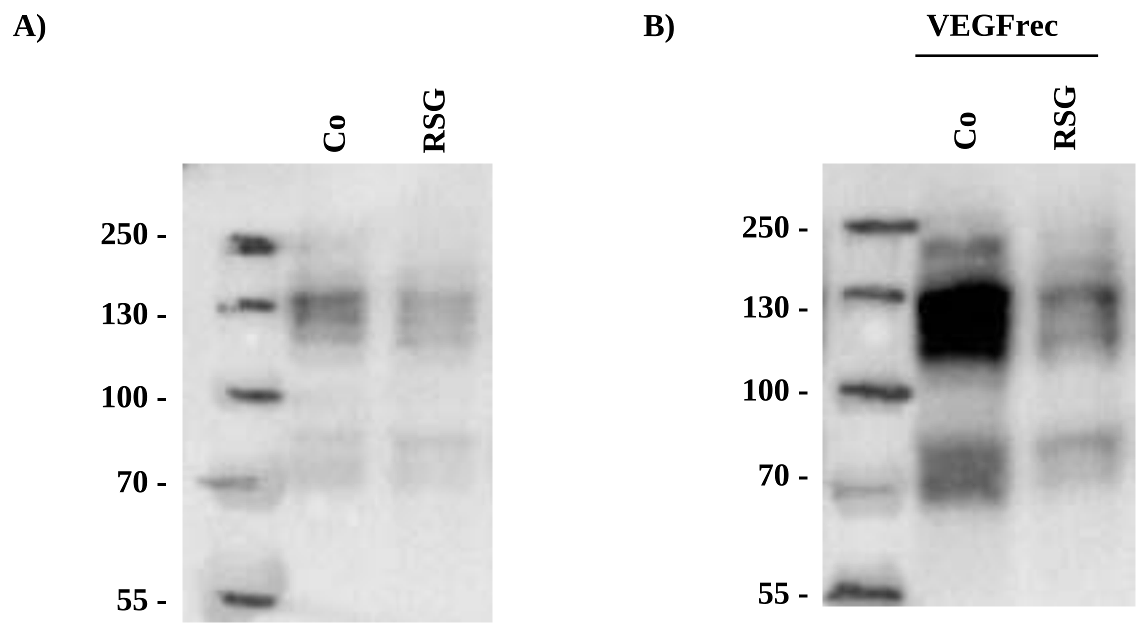

Supplementary Figure S1. RSG decreases expression of global phosphorylation. A) ARPE-19 cells were treated without (Co) or with RSG (12 µM) during 24 h. B) ARPE-19 cells were treated without (Co) or with RSG (12 µM) during 24 h with recombinant VEGF-A at 10 ng/ml during the last 30 minutes of treatment. In A) and B) expression of global phosphorylation with a monoclonal antibody against phospho-tyrosine was assessed by western blotting after 24 h of treatment. Representative blot of phospho-Tyr expression from three independent experiments.

Supplementary Table S1. Antibodies

Antibody against:	Reference #	Providers	Application
β-Actin, clone AC-74	A2228	Sigma Aldrich	Western Blotting
AKT	9272	Sigma Aldrich	
P AKT	4060	Sigma Aldrich	
PARP-1	Sc-8007	Sigma Aldrich	
HSC70	4872	Cell Signaling	
Cav-1	610406	BD	
p Cav-1	611339	BD	
VEGF-R2	2479	Cell Signaling	
pY 1054 VEGF-R2	04-894	Abcam	
pY 951 VEGF-R2	4991	Abcam	
pY1151 VEGF-R2	194806	Abcam	
VEGF-R1	2350	Cell Signaling	
VEGF-R1	2893S	Cell Signaling	
VEGF-A	1316	Cell Signaling	
Ras	3965	Cell Signaling	
p-c-Raf	9421	Cell Signaling	
c-Raf	9422	Cell Signaling	
p-MEK	2338	Cell Signaling	
MEK	9126	Cell Signaling	
p ERK 1/2	9101	Cell Signaling	
ERK 1/2	4695	Cell Signaling	
c-Fos (9F6)	2250	Cell Signaling	
p-Ser 32-cFos	5348	Cell Signaling	
cJun (60A8)	9165	Cell Signaling	
p Ser63-cJun	9261	Cell Signaling	
p Ser73-cJun	3270	Cell Signaling	
VEGF-R2	2479	Cell Signaling	
Cav-1	3238	Cell Signaling	Immunofluorescence (microscopy)
c-Fos (9F6)	2250	Cell Signaling	

cJun (60A8)	9165	Cell Signaling	
p Ser63-cJun	9261	Cell Signaling	
p Ser73-cJun	3270	Cell Signaling	
